# Case Report: Gigantic Arteriovenous Femoral Fistula Following Cardiac Artery Catheterization

**DOI:** 10.3389/fsurg.2022.769302

**Published:** 2022-02-07

**Authors:** Eliza Russu, Adrian Vasile Mureşan, Reka Kaller, Cătălin Mircea Coşarcă, Eliza-Mihaela Arbănaşi, Emil-Marian Arbănaşi

**Affiliations:** ^1^Clinic of Vascular Surgery, Emergency County Hospital, Târgu-Mureş, Romania; ^2^First Department of Surgery, University of Medicine, Pharmacy, Science and Technology “George Emil Palade”, Târgu-Mureş, Romania; ^3^Department of Anatomy, University of Medicine, Pharmacy, Science and Technology “George Emil Palade”, Târgu-Mureş, Romania; ^4^Department F2, Discipline of Pharmaceutical and Therapeutical Chemistry, University of Medicine, Pharmacy, Science and Technology “George Emil Palade”, Târgu-Mureş, Romania

**Keywords:** arteriovenous fistula, vascular injury, vascular surgery, cardiac artery catheterization, iatrogenic arteriovenous fistulas

## Abstract

**Purpose:**

To present the case of a patient with a 9-mm iatrogenic fistula between a branch of the right profunda femoris artery, aneurysmally dilated at ~1.851cm, and the right femoral vein, successfully treated with open surgical ligation.

**Case Report:**

A 70-years-old female was referred to the Vascular Surgery Clinic due to worsening cardiac failure symptoms during the previous year. The medical history included a diagnostic cardiac artery catheterism through a Seldinger technique one year and a half ago. A recent ultrasound described velocities characteristic for a high flow arteriovenous communication in the right groin. Two consecutive cardiology exams, performed at nine months from one another, showed a decrease of almost 21% in the ejection fraction of the left ventricle. An angiography was conducted with the hope of achieving effective percutaneous embolization. Unfortunately, that was not the case. An open repair was scheduled, as the option for a stent-graft deployment was overruled as being too risky, potentially closing several branches of the profunda femoris artery and not fully completing the orifice due to a complicated anatomical positioning. Under local anesthesia, an open ligation was performed in very hostile anatomical conditions. The patient had an uncomplicated evolution and was discharged on the third day, symptom-free.

**Conclusions:**

Iatrogenic arteriovenous fistulas are rare. Clinical presentation diagnoses the case efficiently, vascular imaging being essential for surgical preparation. Though open repair is not the gold standard, there are cases not suitable for the endovascular approach. These patients are eligible for a surgical solution, not without technical challenges.

## Introduction

According to the literature, over 1 million artery catheterizations are conducted in the United States each year with only around 0.001–1% resulting in substantial consequences (1). Radial diagnostic catheterization is much preferable to a femoral approach. Iatrogenic arteriovenous fistulas generally do not present any symptoms and resolve independently without the need for medical intervention (2). In one research including over 10,000 individuals, 38% of cases of iatrogenic femoral arteriovenous fistulas resolved on their own (3).

Femoral iatrogenic AVFs (arteriovenous fistulas) are related to the puncture site, occurring especially after a low puncture, below the femoral bifurcation. They can involve the superficial and deep femoral arteries (4–8).

Post-catheterization AVFs which do not disappear can occasionally grow and manifest with hemorrhage, thrombosis, or even heart failure. This usually will require surgical exploration and repair as it is unlikely to disappear spontaneously, and the fistula may expand as time passes (9).

Direct communication between the arterial and venous puncture sites with active and continuous bleeding from the arterial access site leads to a fistula formation. It is associated with a thrill or specific bruit on examination. Endovascular procedures are not suitable for complex fistulas, as embolization using cyanoacrylate will fail because of the high volume of blood flowing at high velocities through the communication and flushing of the substance. In contrast, a stent graft cannot be placed in any anatomical scenarios.

## Case Presentation

Seventy-years-old woman, referred to our Vascular Surgery Unit functioning within the County Hospital of Targu-Mureş by the Cardiac and Transplantation Unit (IuBCVT) as a chronic patient, dynamic, retired, non-smoking, non-drinking; the patient had been previously transfemoral catheterized (approximately one year prior) through the Seldinger technique, a procedure necessary for a permanent pacemaker (in the VVI mode) implantation. She needed the pacemaker for a third-degree atrioventricular block with an infra-hisian escape rhythm.

We evaluated the patient and found a BMI of 20,5 and mild symptoms of congestive heart failure. The main complaints were fatigue, nocturnal sweating, and tachycardia. The medication prescribed and taken consisted of beta-blockers, SGLT2 inhibitors, ACE inhibitors, and diuretics. Clinical examination objectified a palpable thrill and audible murmur, both characteristic for arteriovenous communication, under and slightly above the right inguinal ligament, with palpable distal pulses. The recorded arterial pressure at admittance was normal, and recent ultrasound examination interpretation had already described velocities characteristic for a high flow arteriovenous communication at the right groin level. A recent echocardiogram revealed moderate mitral insufficiency and a globally impaired left ventricle function, concentrical hypertrophy of the left ventricle, and mild pulmonary hypertension. Two consecutive cardiology exams, performed at nine months from one another, showed a decrease of almost 21% in the ejection fraction of the left ventricle, thus concluding that the cardiac failure is worsening.

**Figure 1:**
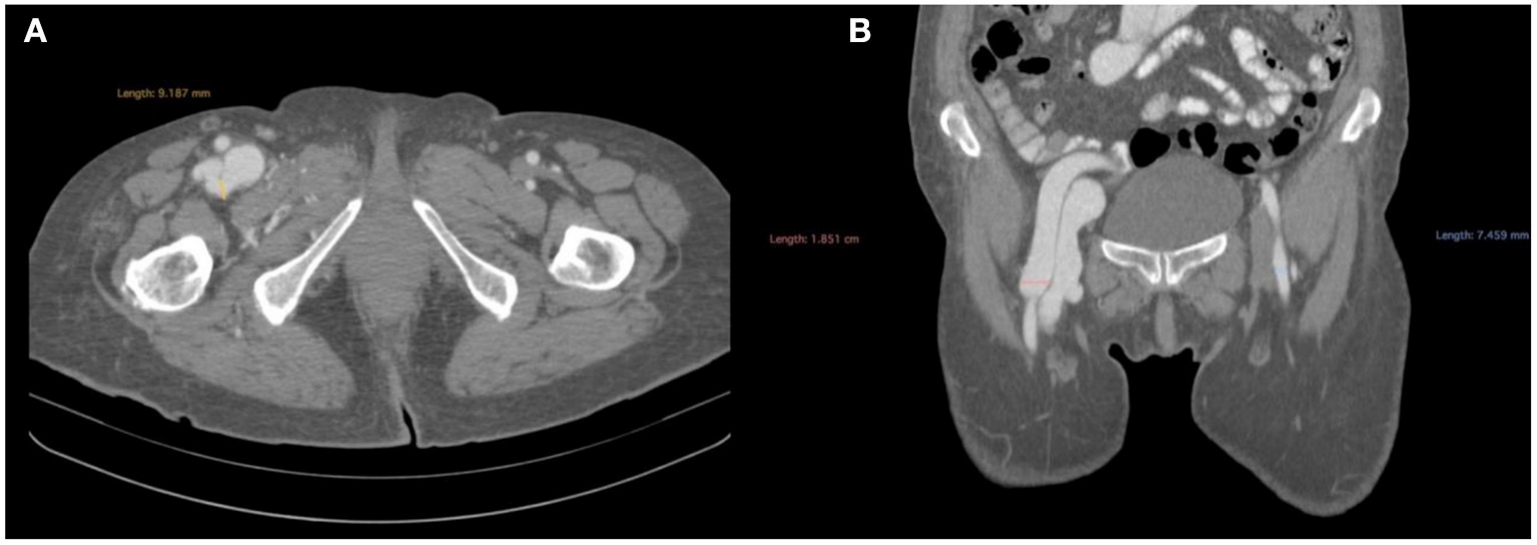
CT angiogprahy before the operation presenting: **(A)** axial section, femoral artery bifurcation visualization and measurement of the fistula and **(B)** coronal section, femoral artery bifurcation- visualization of contrast in the femoral vein.

We repeated the ultrasound examination only to find the femoral vein's high-velocity flow (in the spectral mode), which gained the particularities of an arterial flow with systolic-diastolic pulsations. We then programmed a CT angiography scan, which showed a large fistula, having a 0.9 mm orifice, between the femoral vein (thus grossly dilated) and a branch of the profunda femoris artery (Figure 1).

**Figure 2:**
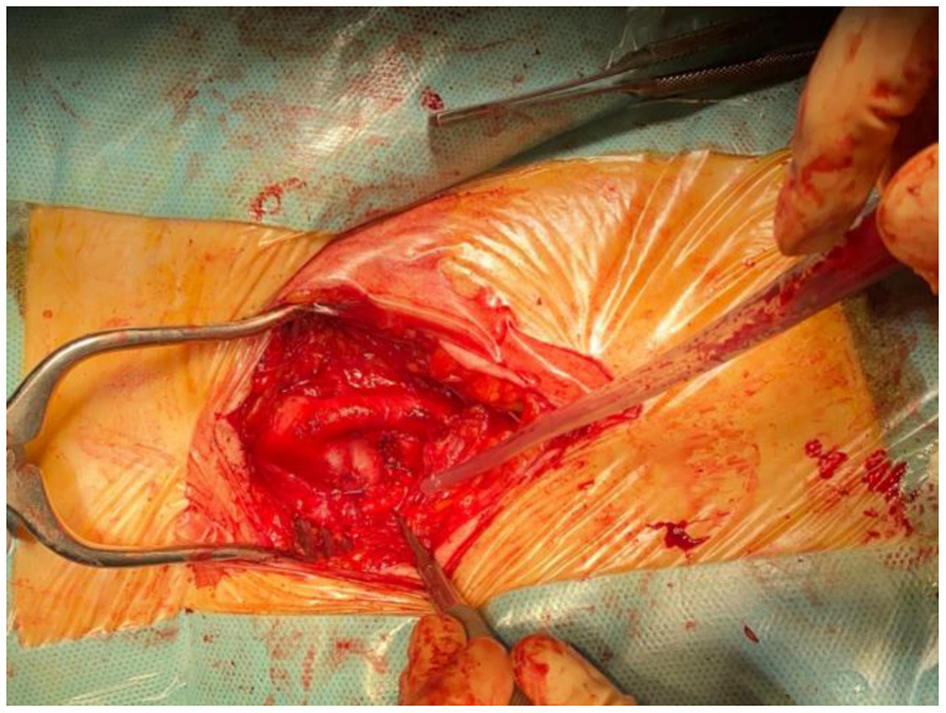
Intraoperative photograph final aspect after the ligation the fistula.

At this point, we admitted the patient. An angiography was conducted in the hope of achieving effective percutaneous embolization. Unfortunately, that was not the case. The option for a stent-graft deployment was overruled as being too risky, potentially closing several branches of the profunda femoris artery and not fully completing the orifice due to a complicated anatomical positioning. Given the rapid deterioration of the heart function, as explained above, we chose an open surgical repair done under local anesthesia (as any other methods implied unjustified risks), with which the patient agreed. The femoral sheet was dissected, and the femoral arteries were prepared and suspended on tourniquets. Systemic heparinization was followed by clamping of the dilated common femoral artery. The tissues between the profunda femoris and the femoral vein were dissected until the fistula appeared in a far posterior approach, resembling a pseudoaneurysm mass vaguely. We then performed a longitudinal arteriotomy on the profunda femoris to better visualize the orifice, as the transverse arteriotomy sheds little light on the area of interest. Once we saw the orifice, we extended the arteriotomy toward a healthy branch of the profunda femoris and cannulated it, performing local heparinization, as well. We proceeded to ligate the fistula using several threads of Surgipro™ Monofilament Polypropylene 5-0., which was a challenging maneuver due to the friability of the “walls” of the fistula. After the successful ligation, we closed the arteriotomy primary, using a continuous suture with Surgipro™ Monofilament Polypropylene 6-0., as the femoral artery was grossly dilated, not requiring a patch to avoid stenosis. After removing the femoral clamp, back-bleeding did not occur. The total clamping time was 20 min. Drainage was unnecessary (Figure 2).

**Figure 3:**
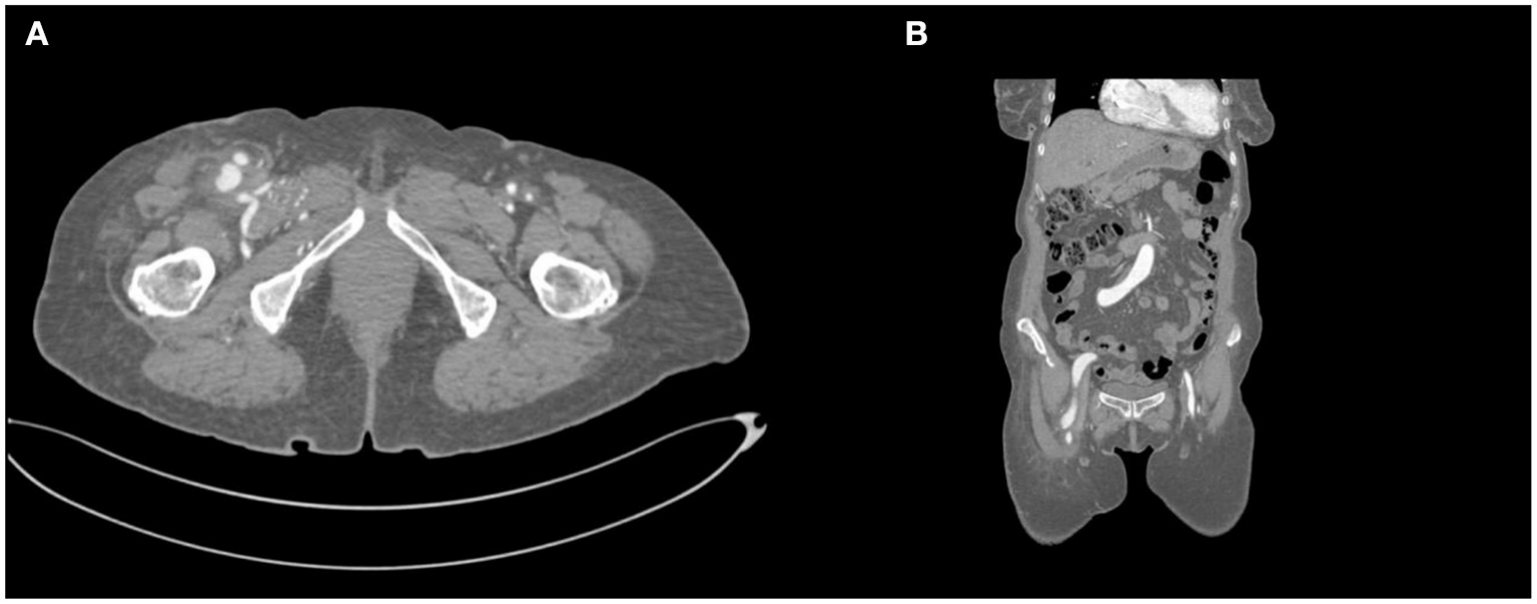
CT angiography after the operation presenting: **(A)** axial section, femoral artery bifurcation lack of contrast in the femoral vein and **(B)** coronal section, femoral artery bifurcation- lack of contrast in the femoral vein.

**Figure 4:**
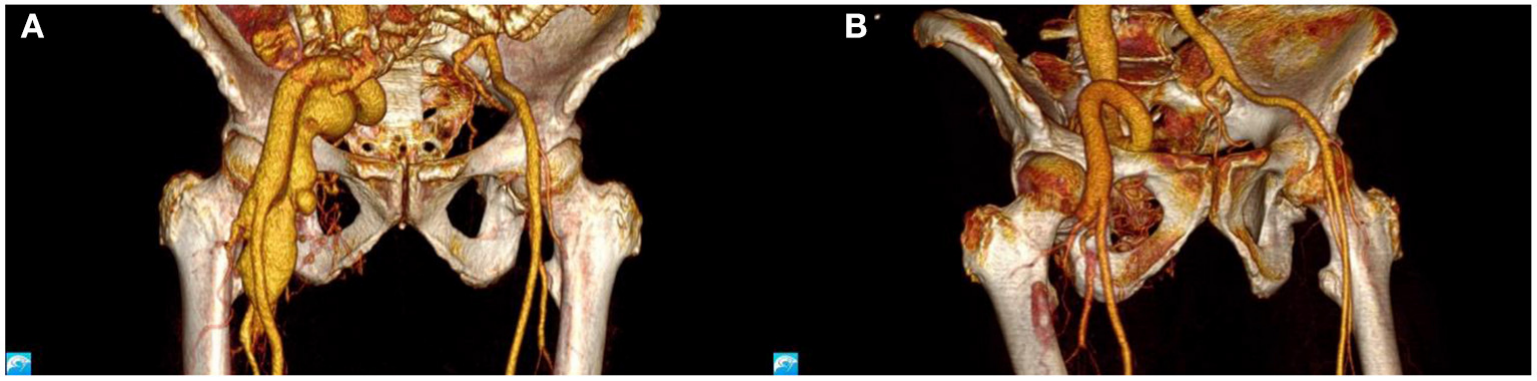
CT angiography, 3D reconstruction: **(A)** before and **(B)** after the operation.

The patient had an uncomplicated postoperative evolution and was discharged on the third day, symptom-free. One month follow-up showed no signs of residual communication, the ultrasonography capturing expected arterial and venous flows. Three months follow-up consisted of a control CT scan, which showed the disappearance of the fistula (Figures 3, 4).

## Discussion

The transfemoral method employing a Seldinger procedure has been regarded as a preferred approach for coronary artery angiography. But, over time, research has shown that a transradial method is much safer and does not have as many complications (10).

The reported incidence of AVFs differs with the diagnostic method, as follows: if ultrasound is used as the primary tool of diagnostic, the incidence is 0.25–2.8% (11–13), clinical detection leads to an incidence of 0.1–0.9% (2, 4, 5, 14–16), and if it is an intraoperative finding then the incidence drops to 0.03%.

The systemic consequences are imitating heart failure, as a left-to-right shunt (which the fistula behaves like) may cause right ventricular volume overload. Some of the patients can manifest with features of right-sided heart failure like dyspnea, lower extremities swelling, orthopnea, and syncope. For this category of patients, the degree of urgency in providing a cure is higher.

As therapeutical approaches, of the first intention, the endovascular repair must be cited, preferred for many advantages, such as shorter hospitalization and the avoiding of the anesthetic and surgical risk (3, 17, 18). In a paper published by Zilinyi et al., a case of a patient presenting with an AVF between the superficial femoral artery and the superficial femoral vein is highlighted, with similar symptomatology to our case. The chosen approach was endovascular, using a stent-graft positioned in the superficial femoral artery (3).

In the paper of Onal et al., ten patients were included, presenting post-catheterization AVFs between the profunda femoris and the femoral vein. Similarly, all cases were solved using endovascular methods, specifically stent-grafts positioned in the profunda femoris (17).

In some selected cases, which are not suitable for endovascular repair, surgery must be considered, the requirements being either ligation or multiple choices of patches, to reconstruct the communicating vessels (9). The anatomical particularities of each case imprint the technical approach. Once the orifice is visualized, the main challenge is to close the communication, as the structures may be highly brittle, and any tearing can cause severe problems in acquiring hemostasis. One limitation in our case was the local anesthesia chosen due to the ASA (American Society of Anesthesiologists) 4 assessment. Should any technical complications occur, local anesthesia's limitations endanger requirements such as remote clamping and extensive tissue dissections.

A large arteriovenous fistula in the groin can be clinically detected easily once the patient is examined. Otherwise, there is no recollection of a particular procedural complication in our patient's medical history, hence the unforeseeable diagnosis. The patient underwent a successful right femoral artery catheterization and cannot remember anything unusual as high-intensity pain or significant bleeding after removal of the catheter. As far as she knows, everything went by the book. So even though there are no data suggesting complications after catheterizations, in some cases, it could be helpful in day-to-day medical practice to clinically examine the puncture sites thoroughly.

## Conclusions

The systemic effects of the arteriovenous fistula are a decrease in both diastolic and systolic pressure, an increase of cardiac output, and slow cardiac hypertrophy. In a patient who already has a pacemaker, these complications justify a rapid resolution of the fistula, but the complicated architecture owing to the fistula's late development and the relatively wide communication opening makes endoluminal techniques ineffective in this situation. The open procedure has to try to protect as many branches of the profunda femoris artery as possible. It is easier to achieve this goal by performing an arteriotomy on the profunda femoris to visualize the fistula.

## Data Availability Statement

The raw data supporting the conclusions of this article will be made available by the authors, without undue reservation.

## Ethics Statement

The studies involving human participants were reviewed and approved by Ethics Committee of the Emergency County Hospital of Târgu-Mureş, Romania. The patients/participants provided their written informed consent to participate in this study.

## Author Contributions

ER and AM established the general design of the study, devised the manuscript, data collection, interpretation of results, and writing the article. El-MA, RK, CC, and Em-MA worked on conception and design of research and interpretation of results.

## Funding

This study was supported by George Emil Palade University of Medicine, Pharmacy, Science and Technology of Târgu-Mureş.

## Conflict of Interest

The authors declare that the research was conducted in the absence of any commercial or financial relationships that could be construed as a potential conflict of interest.

## Publisher's Note

All claims expressed in this article are solely those of the authors and do not necessarily represent those of their affiliated organizations, or those of the publisher, the editors and the reviewers. Any product that may be evaluated in this article, or claim that may be made by its manufacturer, is not guaranteed or endorsed by the publisher.

## References

[1] Thavarajan D Bakran A. Iatrogenic arteriovenous fistula in the groin presenting as cardiac failure. NDT Plus. (2009) 2:46–8. 10.1093/ndtplus/sfn190 25949285 PMC4421475

[2] Kelm M Perings SM Jax T Lauer T Schoebel FC Heintzen MP. Incidence and clinical outcome of iatrogenic femoral arteriovenous fistulas. J Am Coll Cardiol. (2002) 40:291–7. 10.1016/S0735-1097(02)01966-6 12106934

[3] Zilinyi RS Sethi SS Parikh MA Parikh SA. Iatrogenic arteriovenous fistula following femoral access precipitating high-output heart failure. JACC Case Rep. (2021) 3:421–4. 10.1016/j.jaccas.2020.12.031 34317549 PMC8311044

[4] Sidawy AN Neville RF Adib H Curry KM. Femoral arteriovenous fistula following cardiac catheterization: an anatomic explanation. Cardiovasc Surg Lond Engl. (1993) 1:134–7. 8076015 10.1177/096721099300100210

[5] Ohlow M-A Secknus M-A Korn H. von Neumeister A Wagner A Yu J. Incidence and outcome of femoral vascular complications among 18,165 patients undergoing cardiac catheterisation. Int J Cardiol. (2009) 135:66–71. 10.1016/j.ijcard.2008.03.035 18617281

[6] Marsan RE McDonald V Ramamurthy S. Iatrogenic femoral arteriovenous fistula. Cardiovasc Intervent Radiol. (1990) 13:314–6. 10.1007/BF02578634 2124171

[7] Ruebben A Tettoni S Muratore P Rossato D Savio D Rabbia C. Arteriovenous fistulas induced by femoral arterial catheterization: percutaneous treatment. Radiology. (1998) 209:729–34. 10.1148/radiology.209.3.9844666 9844666

[8] Altin R Flicker S Naidech H. Pseudoaneurysm and arteriovenous fistula after femoral artery catheterization: association with low femoral punctures. Am J Roentgenol. (1989) 152:629–31. 10.2214/ajr.152.3.629 2783816

[9] Porter J Al-Jarrah Q Richardson S. A case of femoral arteriovenous fistula causing high-output cardiac failure, originally misdiagnosed as chronic fatigue syndrome. Case Rep Vasc Med. (2014) 2014:e510429. 10.1155/2014/510429 24959370 PMC4055063

[10] Kwac MS Yoon S-J Oh SJ Jeon DW Kim DH Yang JY. Rare case of radial arteriovenous fistula after coronary angiography. Korean Circ J. (2010) 40:677–9. 10.4070/kcj.2010.40.12.677 21267392 PMC3025343

[11] Kresowik TF Khoury MD Miller BV Winniford MD Shamma AR Sharp WJ. A prospective study of the incidence and natural history of femoral vascular complications after percutaneous transluminal coronary angioplasty. J Vasc Surg. (1991) 13:328–35. 10.1016/0741-5214(91)90226-K 1990173

[12] Banfić L Vrkić Kirhmajer M Vojković M Strozzi M Smalcelj A Lasić Z. Access site complications following cardiac catheterization assessed by duplex ultrasonography. Coll Antropol. (2008) 32:385–90. 18756886

[13] Kacila M Vranic H Hadzimehmedagic A Sehovic S Granov N. The frequency of complications of pseudoaneurysms after cardiac interventional diagnostic and therapeutic interventions. Med Arh. (2011) 65:78–81. 21585178

[14] Stegemann E Stegemann B Marx N Lauer T Hoffmann R. Effect of preinterventional ultrasound examination on frequency of procedure-related vascular complications in percutaneous coronary interventions with transfemoral approach. Am J Cardiol. (2011) 108:1203–6. 10.1016/j.amjcard.2011.06.032 21855839

[15] Kent KC McArdle CR Kennedy B Baim DS Anninos E Skillman JJ. prospective study of the clinical outcome of femoral pseudoaneurysma and arteriovenous fistuals induced by arterial puncture. J Vasc Surg. (1993) 17:125–33. 10.1016/0741-5214(93)90016-F 8421328

[16] Perings SM Kelm M Jax T Strauer BE A. prospective study on incidence and risk factors of arteriovenous fistulae following transfemoral cardiac catheterization. Int J Cardiol. (2003) 88:223–8. 10.1016/S0167-5273(02)00400-X 12714202

[17] Önal B Kosar S Gumus T Ilgit ET Akpek S. Postcatheterization femoral arteriovenous fistulas: endovascular treatment with stent-grafts. Cardiovasc Intervent Radiol. (2004) 27:453–8. 10.1007/s00270-004-0176-4 15383847

[18] Liao JL Wang SK Dalsing MC Motaganahalli RL. Endovascular treatment of a persistent traumatic deep femoral arteriovenous fistula after gunshot injury. Vasc Endovascular Surg. (2020) 54:441–4. 10.1177/1538574420918970 32292134

